# Supplementary material for: Clinical Characteristics and Genetic Variants of a Large Cohort of Patients with Retinitis Pigmentosa Using Multimodal Imaging and Next Generation Sequencing
Source: Int J Mol Sci. 2023 Jun 30;24(13):10895. doi: 10.3390/ijms241310895 (PMC10341662; doi:10.3390/ijms241310895)
Supplement: Supplementary file 1 [file ijms-24-10895-s001.zip › ijms-2370890-supplementary.pdf]

Patient #	Gene	Clinical Phenotype	Age at RP symptom onset or diagnosis if known***	Age at which genetic testing was performed	Zygosity	Transcript	Variant(s)			Variant classification per reporting laboratory (ACMG variant classification criteria applied)
218	ADGRV1	Usher syndrome	23	23	compound heterozygous*	NM_032119.3	c.956dup p.(Asn319Lysfs*6)	c.10940del p.(Asn3647Metfs*27)		Pathogenic; Likely Pathogenic
100	AHI1	non-syndromic RP	7	39	compound heterozygous	NM_017651.4	c.1166C>G p.(Ser389*)	c.2582G>A p.(Gly861Glu)		Pathogenic; Likely Pathogenic
42	BBS1	Bardet-Biedl syndrome	9	10	compound heterozygous	NM_024649.4	c.952G>A p.(Gly318Arg)	c.1169T>G p.(Met390Arg)		Likely Pathogenic; Pathogenic
96	BBS1	Bardet-Biedl syndrome	11	11	compound heterozygous	NM_024649.4	c.1169T>G p.(Met390Arg)	c.1100T>A p.(Ile367Asn)		Pathogenic; Likely Pathogenic
159	BBS1	Bardet-Biedl syndrome	12	13	homozygous	NM_024649.4	c.1169T>G p.(Met390Arg)			Pathogenic
9	BBS2**	Bardet-Biedl syndrome	5	2	compound heterozygous	NM_031885.5	c.72C>G p.(Tyr24*)	c.1438C>T p.(Arg480*)		Pathogenic; Pathogenic
157	CDHR1	non-syndromic RP	18	26	homozygous	NM_033100.3	c.1485+2T>G p.(?)			Pathogenic
67	CERKL	non-syndromic RP	<32	37	compound heterozygous*	NM_001030311.2	c.375C>G p.(Cys125Trp)	c.193G>T p.(Glu65*)		Pathogenic; Pathogenic
86	CERKL	non-syndromic RP	<30	30	compound heterozygous*	NM_001030311.2	c.193G>T p.(Glu65*)	c.375C>G p.(Cys125Trp)		Pathogenic; Likely Pathogenic
80	CLN3	neuronal ceroid lipofuscinosis typ	18	18	homozygous	NM_001042432.1	c.125+1G>C p.(?)			Pathogenic
47	CLRN1	Usher syndrome	5	26	homozygous	NM_174878.2	c.149_152delinsTGTCCAAT p.(Ser50Leufs*12)			Pathogenic
54	CNGA1	non-syndromic RP	<29	29	compound heterozygous	NM_000087.3	c.117C>A p.(Cys39*)	c.1547T>G p.(Met516Arg)		Pathogenic; Variant of uncertain significance
114	EYS	non-syndromic RP	36	45	compound heterozygous*	NM_001142800.1	c.6269G>A p.(Trp2090*)	c.6714delT p.(Ile2239Serfs*17)		Pathogenic; Pathogenic
146	EYS	non-syndromic RP	52	60	compound heterozygous*	NM_001142800.1	c.9036del p.(Leu3013Serfs*6)	c.8255_8260del p.(Leu2752_Asn2754delinsTyr)		Pathogenic; Variant of uncertain significance
171	EYS	non-syndromic RP	26	64	compound heterozygous*	NM_001142800.1	c.32dup p.(Met12Aspfs*14)	c.4350_4356del p.(Ile145Profs*3)		Pathogenic; Pathogenic
222	EYS	non-syndromic RP	19	59	compound heterozygous*	NM_001142800.1	c.1155T>A p.(Cys385*)	c.(6424+1_6425-1)_(6725+1_6726-1)del p.(Asp2142Alafs*14)		Pathogenic; Pathogenic
122	FSCN2	non-syndromic RP	6	14	heterozygous	NM_012418.3	c.731C>T p.(Thr244Met)			Variant of uncertain significance
27	GUCA1A	non-syndromic RP	<27	27	heterozygous	NM_000409.4	c.464A>G p.(Glu155Gly)			Pathogenic
87	GUCY2D	non-syndromic RP	7	11	heterozygous	NM_000180.3	c.2513G>A p.(Arg838His)			Pathogenic
172	IDH3A	non-syndromic RP	7	45	compound heterozygous*	NM_005530.2	c.946C>T p.(Arg316Cys)	c.533G>A p.(Arg178His)		Likely pathogenic; Variant of uncertain significance
153	IMPG2	non-syndromic RP	13	23	compound heterozygous*	NM_016247.4	c.3262C>T p.(Arg1088*)	c.1263G>A p.(Trp421*)		Pathogenic; Likely Pathogenic
174	KLHL7	non-syndromic RP	15	29	heterozygous	NM_001031710.2	c.458C>T p.(Ala153Val)			Pathogenic
221	MAK	non-syndromic RP	41	44	homozygous	NM_001242957.2	c.911_914del p.(Asn304Serfs*6)			Pathogenic
150	MAP2K2	cardiofaciocutaneous syndrome	17	17	heterozygous	NM_030662.4	c.183A>C p.(Lys61Asn)			Pathogenic
113	MERTK	non-syndromic RP	7	25	compound heterozygous*	NM_006343.2	c.(?_-122)_(1144+1_1145-1)del	c.2180G>A p.(Arg727Gln)		Pathogenic; Pathogenic
134	MFRP	non-syndromic RP	60	62	compound heterozygous*	NM_031433.3	c.734G>T p.(GLy245Val)	c.1124+1G>A p.(?)		Variant of uncertain significance; Likley Pathogenic
110	MYO7A	Usher syndrome	7	53	compound heterozygous*	NM_000260.3	c.5968C>T p.(Gln1990*)	c.4442-2A>C p.(?)		Pathogenic; Likely Pathogenic
152	MYO7A	Usher syndrome	19	20	compound heterozygous*	NM_000260.3	c.1003+1_1003+33delinsCAGTGCCTTG p.(?)	c.1595A>G (p.His532Arg)		Likely pathogenic; Variant of uncertain significance
196	MYO7A	Usher syndrome	3	7	compound heterozygous*	NM_000260.3	c.582delC p.(Phe195fs*)	c.5886_5888delCTT p.(Phe1963del)		Pathogenic; Pathogenic
197	MYO7A	Usher syndrome	6	4	compound heterozygous*	NM_000260.3	c.582delC p.(Phe195fs*)	c.5886_5888delCTT p.(Phe1963del)		Pathogenic; Pathogenic
200	MYO7A	Usher syndrome	10	65	homozygous	NM_000260.3	c.1996C>T (p.Arg666*)			Pathogenic
202	MYO7A	Usher syndrome	6	7	compound heterozygous*	NM_000260.3	c.1900C>T p.(Arg634X),	c.1976C>A p.(Ser659X),		Pathogenic; Pathogenic
207	MYO7A	Usher syndrome	8	4	compound heterozygous*	NM_000260.3	c.3476G>T p.(Gly1159Val)	c.5392C>T p.(Gln1798*)		Pathogenic; Pathogenic
208	MYO7A	Usher syndrome	15	37	compound heterozygous*	NM_000260.3	c.3719G>A p.(Arg1240Gln)	c.2838del p.(Met946Ilefs*116)		Pathogenic, Pathogenic
213	MYO7A	Usher syndrome	24	41	compound heterozygous*	NM_000260.3	c.494C>T p.(Thr165Met)	c.1349A>T p.(Glu450Val)		Pathogenic; Likely Pathogenic
215	MYO7A	Usher syndrome	<38	49	compound heterozygous*	NM_000260.3	c.3719G>A p.(Arg1240Gln)	c.6439-2A>G p.(?)		Pathogenic; Pathogenic
213	MYO7A	Usher syndrome	14	40	compound heterozygous*	NM_000260.3	c.494C>T p.(Thr165Met)	c.1349A>T p.(Glu450Val)		Pathogenic; Likely Pathogenic
147	NPHP1	nephronophthisis with RP	46	61	homozygous	NM_000272.3	c.(69+1_70-1)_(*455_?)del			Pathogenic
31	NR2E3	non-syndromic RP	11	53	homozygous	NM_014249.3	c.932G>A p.(Arg311Gln)			Pathogenic
148	PCARE	non-syndromic RP	66	75	compound heterozygous*	NM_001029883.2	c.2950C>T p.(Arg984*)	c.3521_3538del (p.Asp1174_Ala1179del)		Pathogenic; Variant of uncertain significance
14	PDE6B	non-syndromic RP	16	51	compound heterozygous*	NM_000283.3	c.2193+1G>A p.(?)	c.1765dup p.(Ala589Glyfs*10)		Pathogenic; Pathogenic
49	PDE6B	non-syndromic RP	11	11	compound heterozygous	NM_000283.3	c.1466T>C p.(Leu489Pro)	c.1928_1968del41 p.(Ile644fs*)		Variant of uncertain significance ("probably damaging"); Likely Pathogenic
5	POMGNT1	muscular dystrophy-dystroglycan	40	46	compound heterozygous	NM_017739.3	c.1539+1G>A p.(?)	c.1453C>T p.(Arg485Cys)		Pathogenic; Likely Pathogenic
77	PRPF3	non-syndromic RP	10	10	heterozygous	NM_004698.2	c.1481C>T p.(Thr49Met)			Pathogenic
93	PRPF31	non-syndromic RP	23	40	heterozygous	NM_015629.3	c.239-1G>A p.(?)			Pathogenic
17	PRPH2	non-syndromic cone-rod dystropl	50	61	heterozygous	NM_000322.4	c.811_813del p.(Leu271del)			Likely Pathogenic
18	PRPH2	non-syndromic cone-rod dystropl	50	51	heterozygous	NM_000322.4	c.811_813del p.(Leu271del)			Likely Pathogenic
20	PRPH2	non-syndromic cone-rod dystropl	43	58	heterozygous	NM_000322.4	c.811_813del p.(Leu271del)			Likely Pathogenic
12	RHO	non-syndromic RP	18	52	heterozygous	NM_000539.3	c.1040C>G p.(Pro347Arg)			Pathogenic
89	RHO**	non-syndromic RP	10	63	heterozygous	NM_000539.3	c.173C>G p.(Thr58Arg)			Pathogenic
16	RLBP1	non-syndromic RP	20	55	homozygous	NM_000326.4	c.(525+1_526-1)_(*418_?)del			Pathogenic
37	RP1	non-syndromic RP	<60	69	heterozygous	NM_006269.1	c.2321_2322insAlu p.(Leu774fs)			Pathogenic
56	RP1	non-syndromic RP	23	28	heterozygous	NM_006269.1	c.2219C>A p.(Ser740*)			Pathogenic
154	RP1	non-syndromic RP	41	43	heterozygous	NM_006269.1	c.2321_2322ins? p.(Leu774fs)			Pathogenic
178	RP1	non-syndromic RP	<30	39	homozygous	NM_006269.1	c.4804C>T p.(Gln1602*)			Pathogenic
32	RPGR	non-syndromic RP	22	59	hemizygous	NM_001034853.1	c.2384del p.(Glu795Glyfs*20)			Pathogenic
36	RPGR	non-syndromic RP	6	13	hemizygous	NM_001034853.1	c.2568dup p.(Lys857Glufs*222)			Pathogenic
73	RPGR	non-syndromic RP	4	9	hemizygous	NM_001034853.1	c.2384del p.(Glu795Glyfs*20)			Pathogenic
128	RPGR	non-syndromic RP	<18	27	hemizygous	NM_001034853.1	c.2405_2406del p.(Glu802Glyfs*32)			Pathogenic
131	RPGR	non-syndromic RP	39	39	heterozygous	NM_001034853.1	c.2505_2506delGG p.(Glu836Argfs*242)			Pathogenic
132	RPGR	non-syndromic RP	8	9	hemizygous	NM_001034853.1	c.2505_2506delGG p.(Glu836Argfs*242)			Pathogenic
135	RPGR	non-syndromic RP	22	51	hemizygous	NM_001034853.1	c.2426_2427del p.(Glu809Glyfs*25)			Pathogenic
167	RPGR	non-syndromic RP	4	4	hemizygous	NM_001034853.1	c.2426_2427del p.(Glu809Glyfs*25)			Pathogenic
168	RPGR	non-syndromic RP	ently asymptomatic	3	hemizygous	NM_001034853.1	c.2426_2427del p.(Glu809Glyfs*25)			Pathogenic
169	RPGR	non-syndromic RP	ently asymptomatic	2	hemizygous	NM_001034853.1	c.2426_2427del p.(Glu809Glyfs*25)			Pathogenic
223	RPGR	non-syndromic RP	14	29	hemizygous	NM_001034853.1	c.2323_2324del p.(Arg775Glufs*59)			Pathogenic
156	RPGRIP1	non-syndromic RP	22	75	compound heterozygous*	NM_020366.3	c.2718dup p.(Asn907*)	c.(3099+1_3100-1)_(3238+1_3239-1)del		Pathogenic; Pathogenic
61	RS1	non-syndromic RP	<57	57	hemizygous	NM_000330.3	c.214G>A p.(Glu72Lys)			Pathogenic
8	USH2A	non-syndromic RP	29	36	compound heterozygous	NM_206933.2	c.2276G>T p.(Cys759Phe)	c.4645C>T p.(Arg1549*)		Pathogenic; Pathogenic
46	USH2A	non-syndromic RP	<70	70	compound heterozygous*	NM_206933.2	c.7524del p.(Arg2509Glyfs*19)	c.2276G>T p.(Cys759Phe)		Pathogenic; Likely Pathogenic
65	USH2A	non-syndromic RP	<18	57	compound heterozygous*	NM_206933.2	c.11074del p.(Ile3692Serfs*26)	c.14203C>T p.(Pro4735Ser)		Pathogenic; Variant of uncertain significance
193	USH2A	Usher syndrome	16	22	compound heterozygous*	NM_206933.2	c.11864G>A p.(Trp3955*)	c.(4627+1_4628-1)_(6049+1_6050-1)del		Pathogenic; Pathogenic
194	USH2A	Usher syndrome	24	44	compound heterozygous*	NM_206933.2	c.2276G>T (p.Cys759Phe)	c.5506C>A (p.Pro1836Thr)	c.13778C>T p.(Ser4593Leu)	Pathogenic; Pathogenic; Variant of uncertain significance
198	USH2A	Usher syndrome	50	61	compound heterozygous	NM_206933.2	c.7595-3C>G p.(?)	c.4106C>T p.(Ser1369Leu)		Pathogenic; Likey Pathogenic
203	USH2A	Usher syndrome	14	63	compound heterozygous*	NM_206933.2	c.9885T>G (p.Cys3295Trp)	c.14792-2A>G p.(?)		Pathogenic; Pathogenic
204	USH2A	Usher syndrome	37	72	compound heterozygous*	NM_206933.2	c.14791+2T>A p.(?)	c.(2809+1_2810-1)_(7120+1_7121-1)dup		Pathogenic; Variant of uncertain significance
210	USH2A	non-syndromic RP	64	61	compound heterozygous	NM_206933.2	c.11864G>A p.(Trp3955*)	c.12268C>A p.(Pro4090Thr)		Pathogenic; Pathogenic
211	USH2A	non-syndromic RP	65	65	compound heterozygous*	NM_206933.2	c.2332G>T p.(Asp778Tyr)	c.13349C>T p.(Pro4450Leu)		Pathogenic; Likely Pathogenic
219	USH2A	non-syndromic RP	40	40	compound heterozygous*	NM_206933.2	c.10561T>C p.(Trp3521Arg)	c.8981G>A p.(Trp2994*)		Pathogenic; Pathogenic
255	USH2A	non-syndromic RP	67	86	compound heterozygous*	NM_206933.2	c.2276G>T p.(Cys759Phe)	c.920_923dup p.(His308Glnfs*16)		Pathogenic; Pathogenic
33	USH2A	non-syndromic RP	37	38	compound heterozygous*	NM_206933.2	c.2276G>T p.(Cys759Phe)	c.5516T>A p.(Val1839Glu)		Pathogenic; Pathogenic
216	USH2A**	Usher syndrome	26	35	homozygous	NM_206933.2	c.7595-2144A>G p.(?)			Pathogenic
25	VPS13B	Cohen syndrome	8	16	compound heterozygous	NM_01789.03	c.6002delC p.(Pro2001Leufs*18)	c.11314C>T p.(Gln3772*)		Pathogenic; Pathogenic
182	VSP13B	Cohen syndrome	13	24	compound heterozygous	NM_01789.03	c.6733-1G>A p.(?)	c.11119G>A p.(?)		Pathogenic; Likely Pathogenic

* two variants presumed to be in trans (no family member testing performed to confirm and/or data not available from the lab to confirm trans configuration)

**variants per clinical documentation only

***age of onset or diagnosis is per chart review and patient report
